# Supplementary figures and images for: Demographic history and genomics of local adaptation in blue tit populations
Source: Evol Appl. 2020 Jul 14;13(6):1145–65. doi: 10.1111/eva.13035 (PMC7359843; doi:10.1111/eva.13035)

Supplementary Figure 1. 5 PCA between i) all the individuals, ii) A2D vs A2E, iii) B4D vs B4E, iv) B5D vs B5E, v) B6D vs B6E

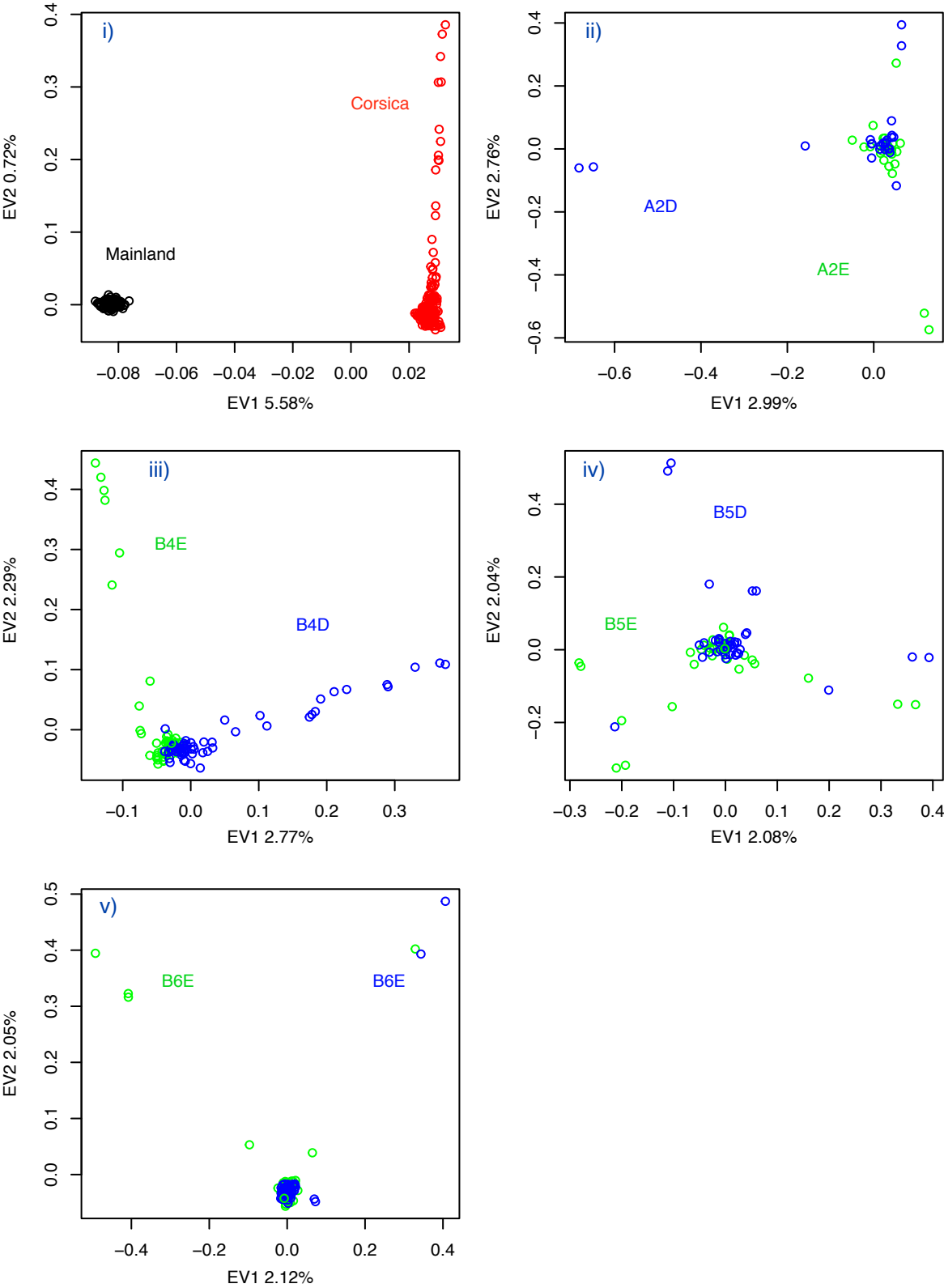

Supplement: Supplementary file 1 — Fig S1 [file EVA-13-1145-s001.pdf]

Supplementary Figure 7. Total length of ROH as a function of total number of ROH, per population.

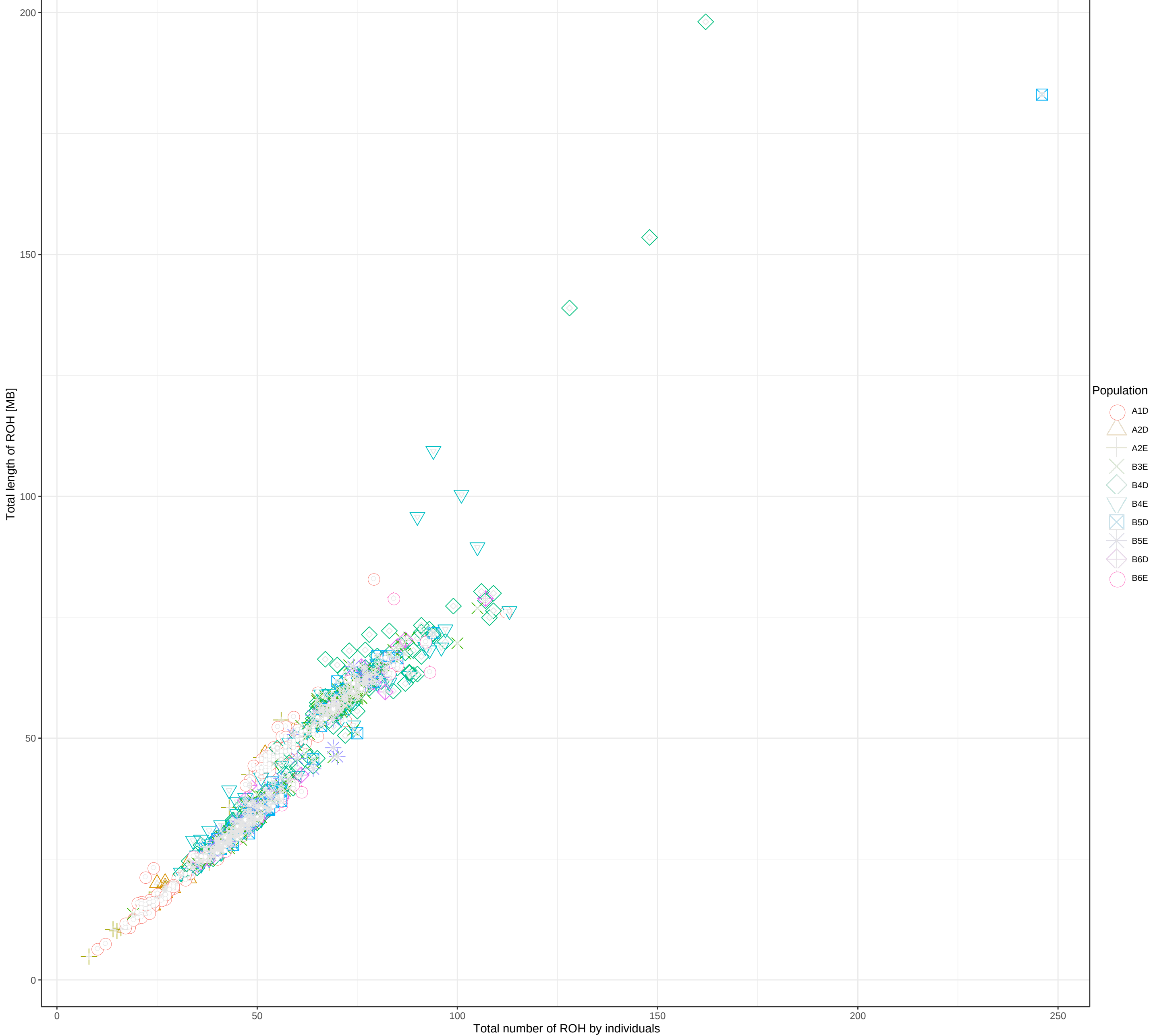

Supplement: Supplementary file 7 — Fig S7 [file EVA-13-1145-s007.pdf]

Supplementary Figure 8. Boxplot of ROH length (Kb) per population

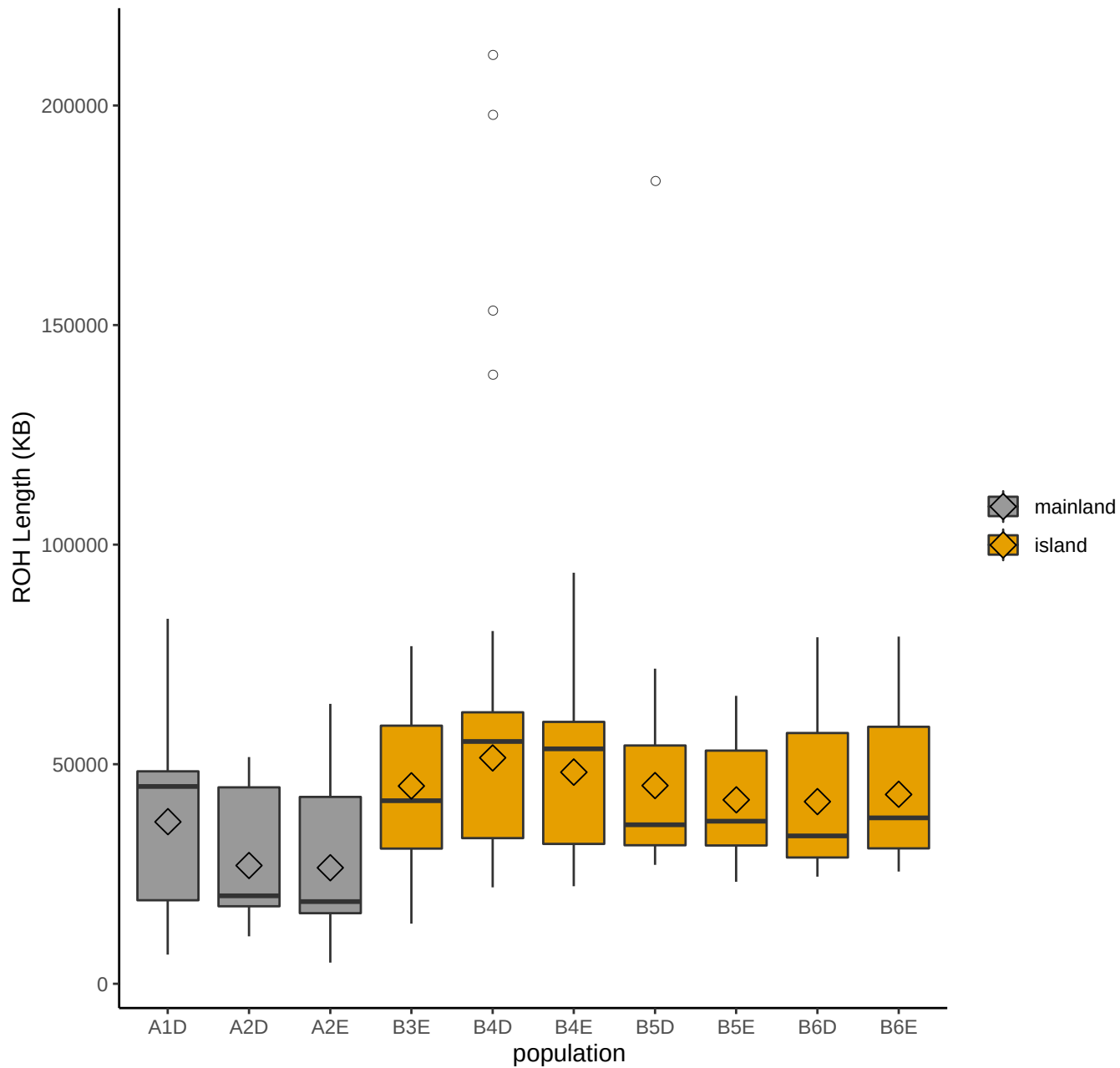

Supplement: Supplementary file 8 — Fig S8 [file EVA-13-1145-s008.pdf]

Supplementary Figure 11.  $F_{ST}$  Corsica-Mainland  
as a function of recombination rate

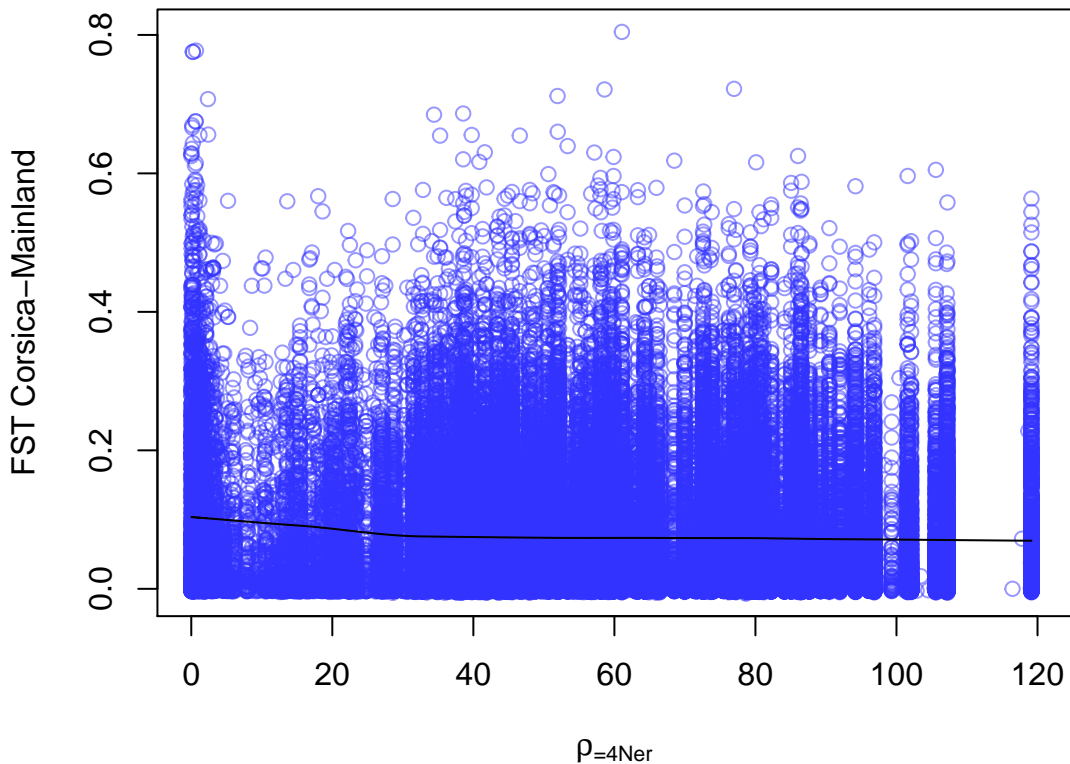

Supplement: Supplementary file 11 — Fig S11 [file EVA-13-1145-s011.pdf]
