# Supplementary material for: Demographic history and genomics of local adaptation in blue tit populations
Source: Evol Appl. 2020 Jul 14;13(6):1145–65. doi: 10.1111/eva.13035 (PMC7359843; doi:10.1111/eva.13035)

Supplementary Figure 2: Prior and posterior probabilities of estimated parameters for each pair of population.

Theta1 = 4N1/µ (scale mutation rate for the population 1), Theta2 = 4N2/µ (scaled mutation rate for population 2). ThetaA = 4Nanc/µ/. (scaled mutation rate for the ancestral population. All theta are scale by the theta of the reference population (4*Nref*µ). Migration rate M_{ij} = 4N * ref *m_{ij}. . Split time for the IM model is scaled by the 4Nref so that τ =Tsplit/4Nref.

A2DE – Best model = Isolation With Migration

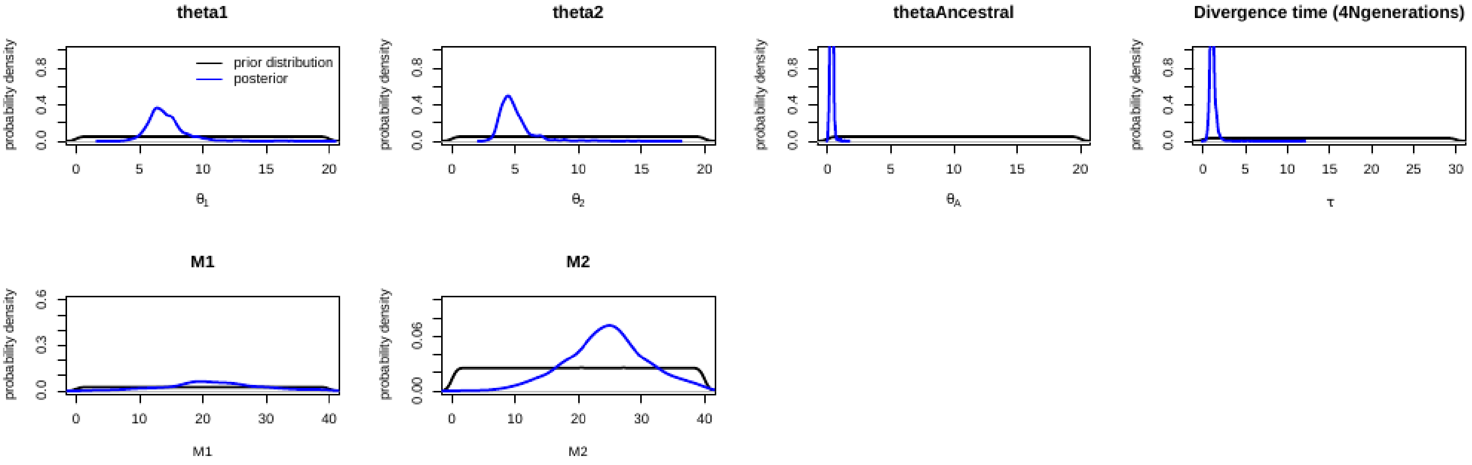

A2DE – Second best model: = Equilibrium (Island model)

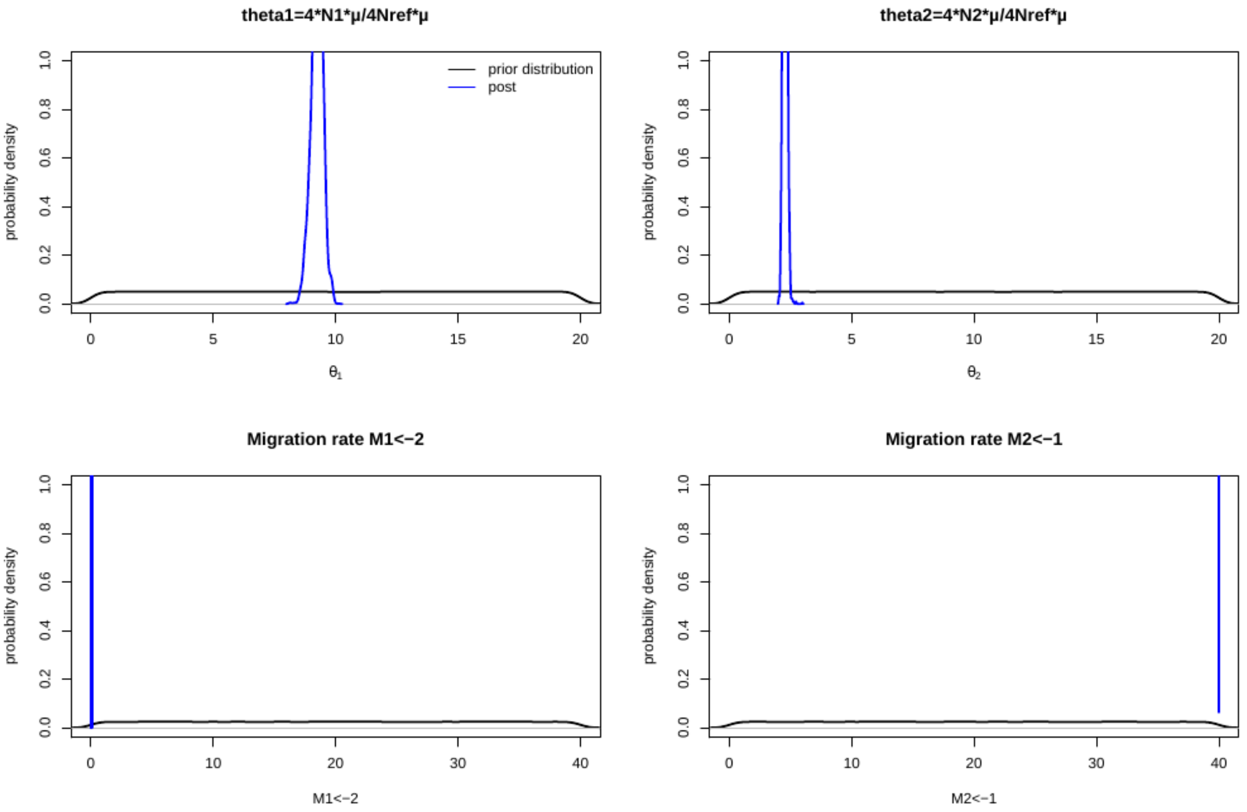

B4DE – Best model = Equilibrium (Island model)

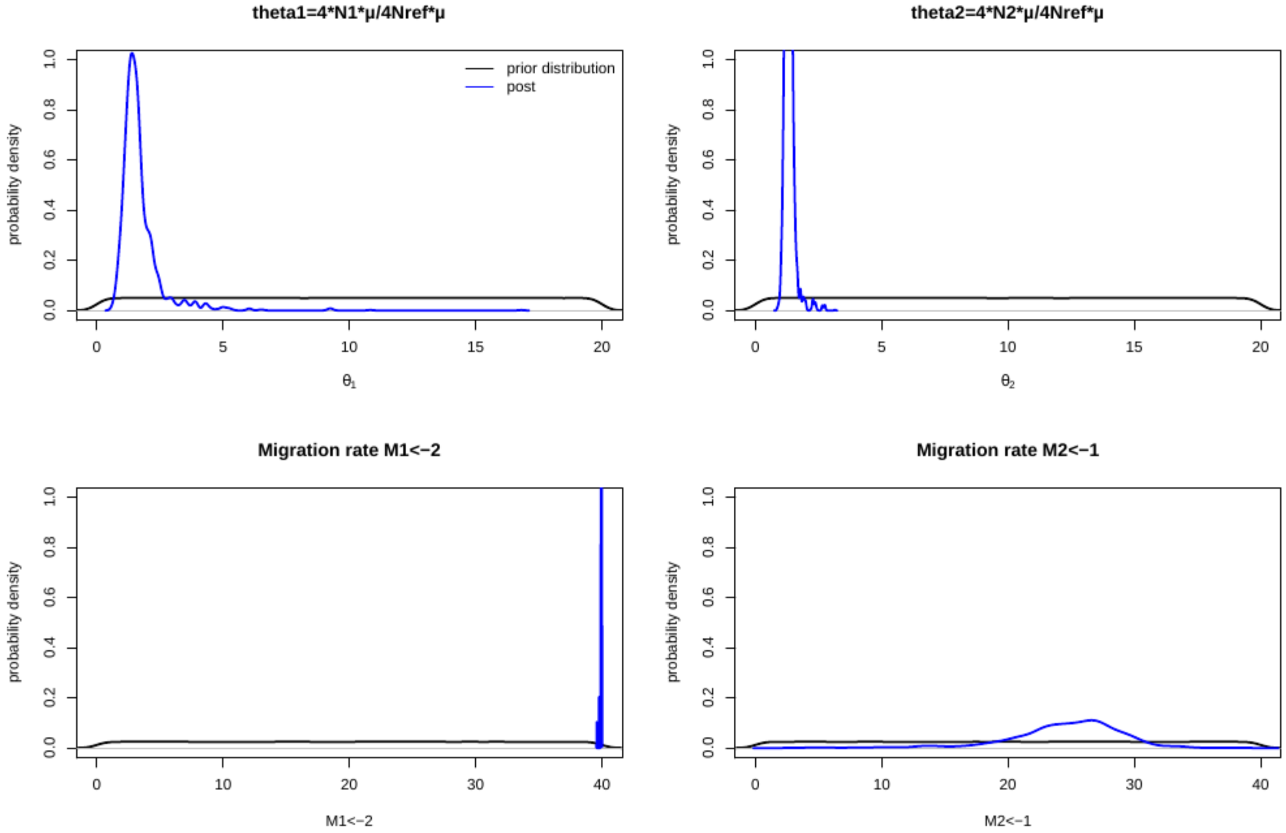

B5DE – Best model = Equilibrium (Island model)

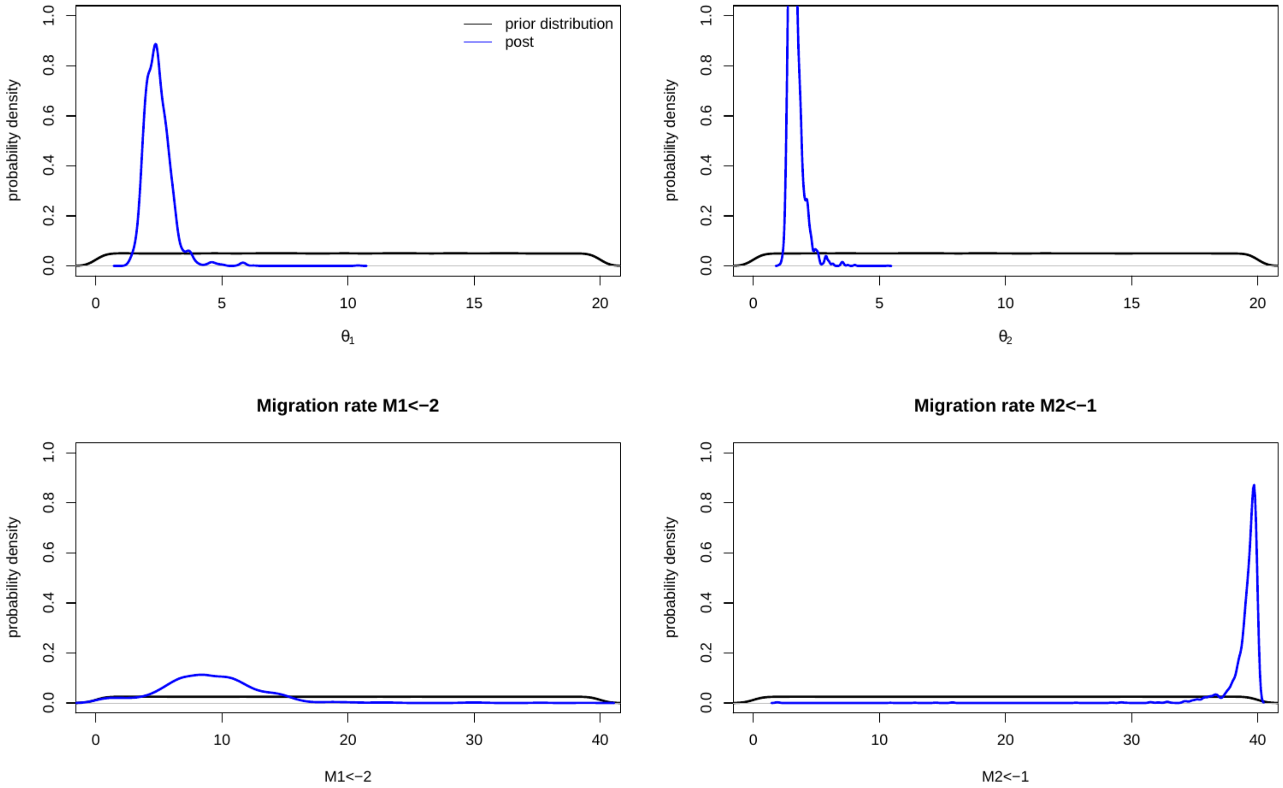

B6DE – Best model = Equilibrium (Island model)

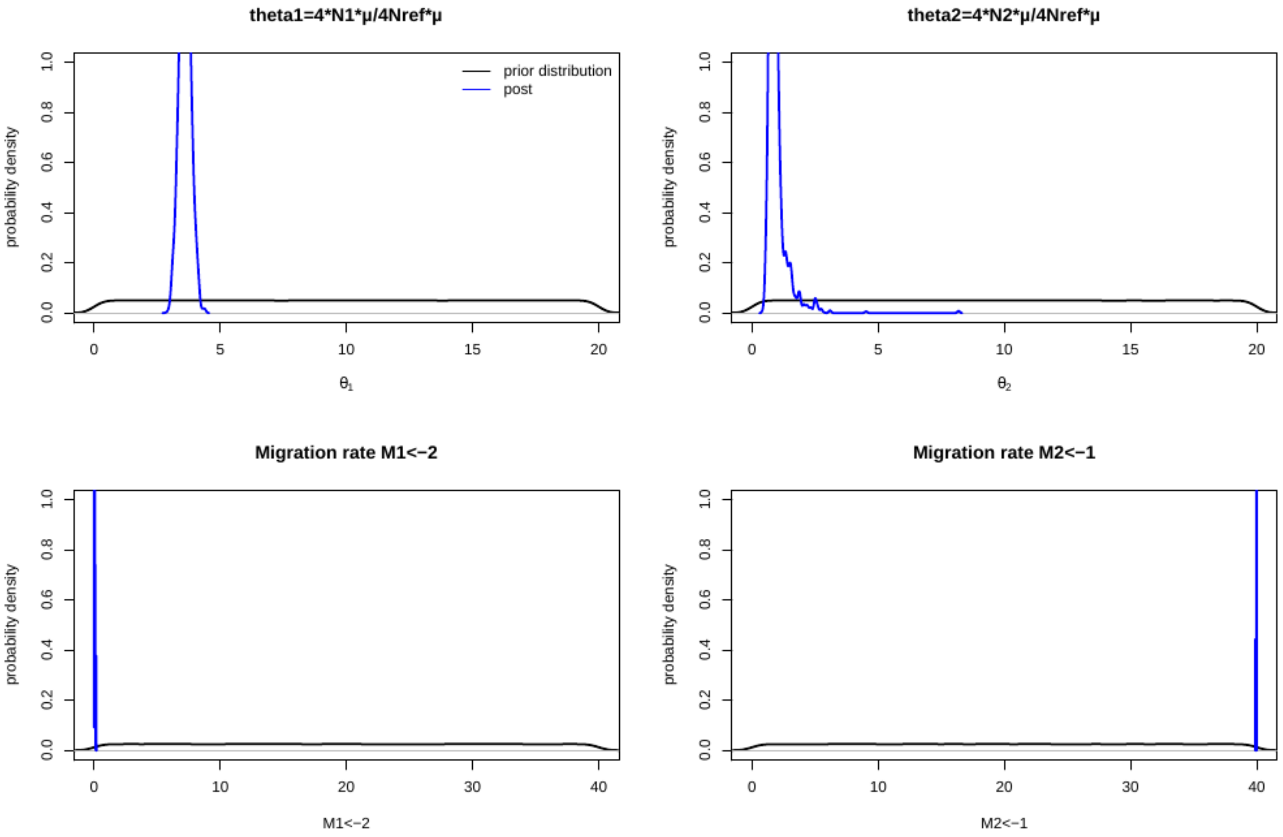

Supplement: Supplementary file 2 — Fig S2 [file EVA-13-1145-s002.pdf]
