# Supplementary material for: Demographic history and genomics of local adaptation in blue tit populations
Source: Evol Appl. 2020 Jul 14;13(6):1145–65. doi: 10.1111/eva.13035 (PMC7359843; doi:10.1111/eva.13035)
Supplement: Supplementary file 4 — Fig S4 [file EVA-13-1145-s004.pdf]

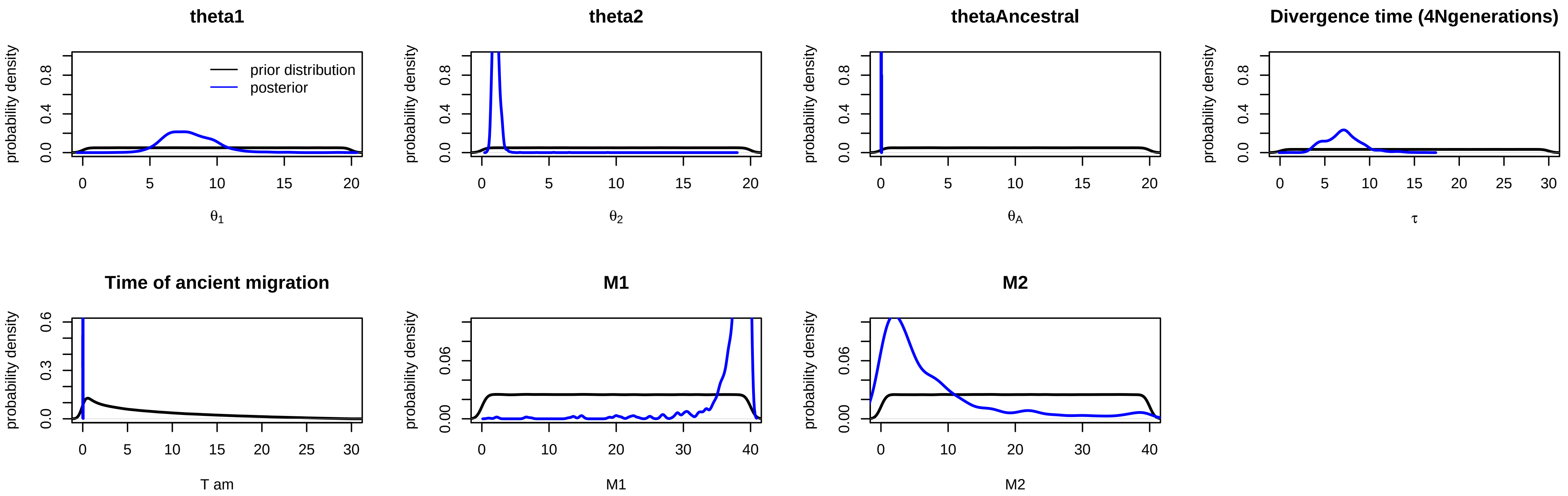

Supplementary Figure 4. Prior and posterior probabilities for models infering mainland-Corsica divergence
