# Supplementary material for: Demographic history and genomics of local adaptation in blue tit populations
Source: Evol Appl. 2020 Jul 14;13(6):1145–65. doi: 10.1111/eva.13035 (PMC7359843; doi:10.1111/eva.13035)

Supplementary Figure 5. A) MAF histogram, B) LD decay with genomic distance (bp), and C) temporal Ne, for each populations.

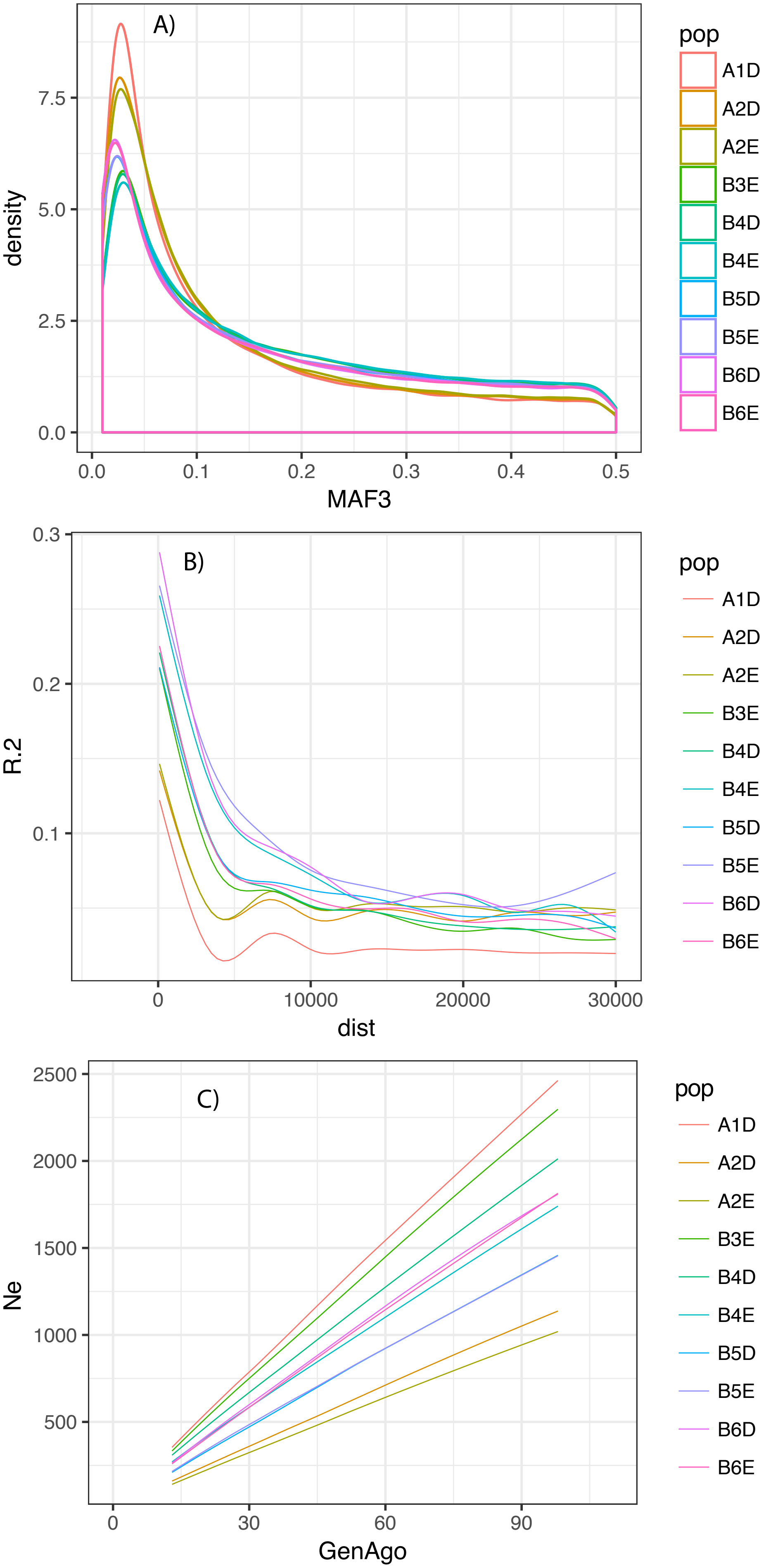

Supplement: Supplementary file 5 — Fig S5 [file EVA-13-1145-s005.pdf]
