# Supplementary material for: Demographic history and genomics of local adaptation in blue tit populations
Source: Evol Appl. 2020 Jul 14;13(6):1145–65. doi: 10.1111/eva.13035 (PMC7359843; doi:10.1111/eva.13035)

Supplementary Figure 6. LD decay with genomic distance, for each populations, for the chromosomes 1, 2 and Z.

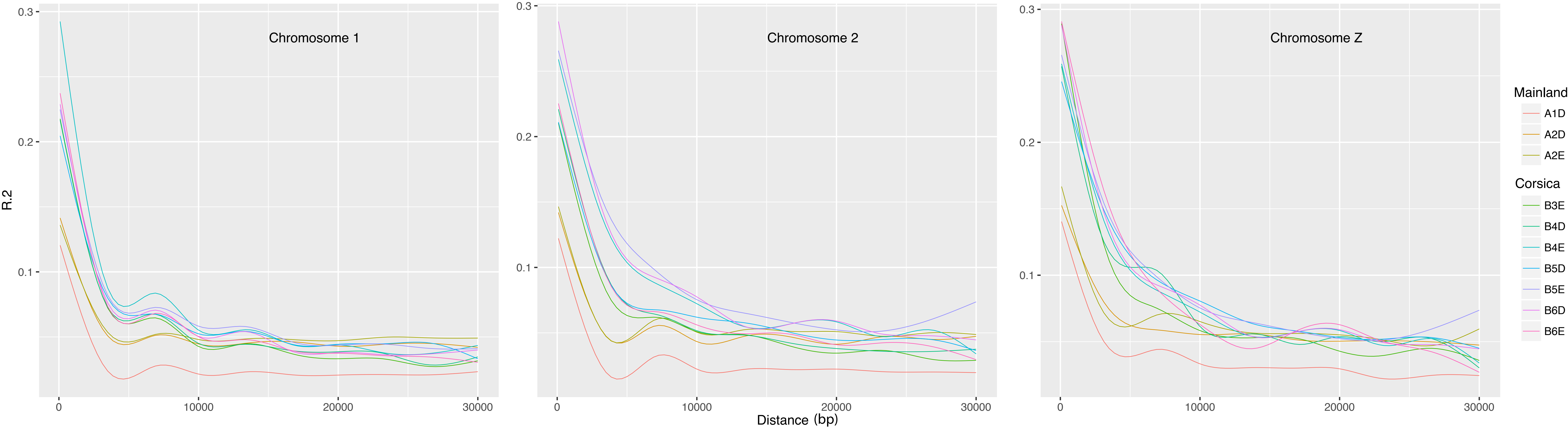

Supplement: Supplementary file 6 — Fig S6 [file EVA-13-1145-s006.pdf]
