# Supplementary material for: Demographic history and genomics of local adaptation in blue tit populations
Source: Evol Appl. 2020 Jul 14;13(6):1145–65. doi: 10.1111/eva.13035 (PMC7359843; doi:10.1111/eva.13035)

Supplementary Figure 9. Fst as a function of Bayescan log10(BF) outputs for each SNP, for i) Corsica vs mainland and ii) evergreen vs deciduous populations.

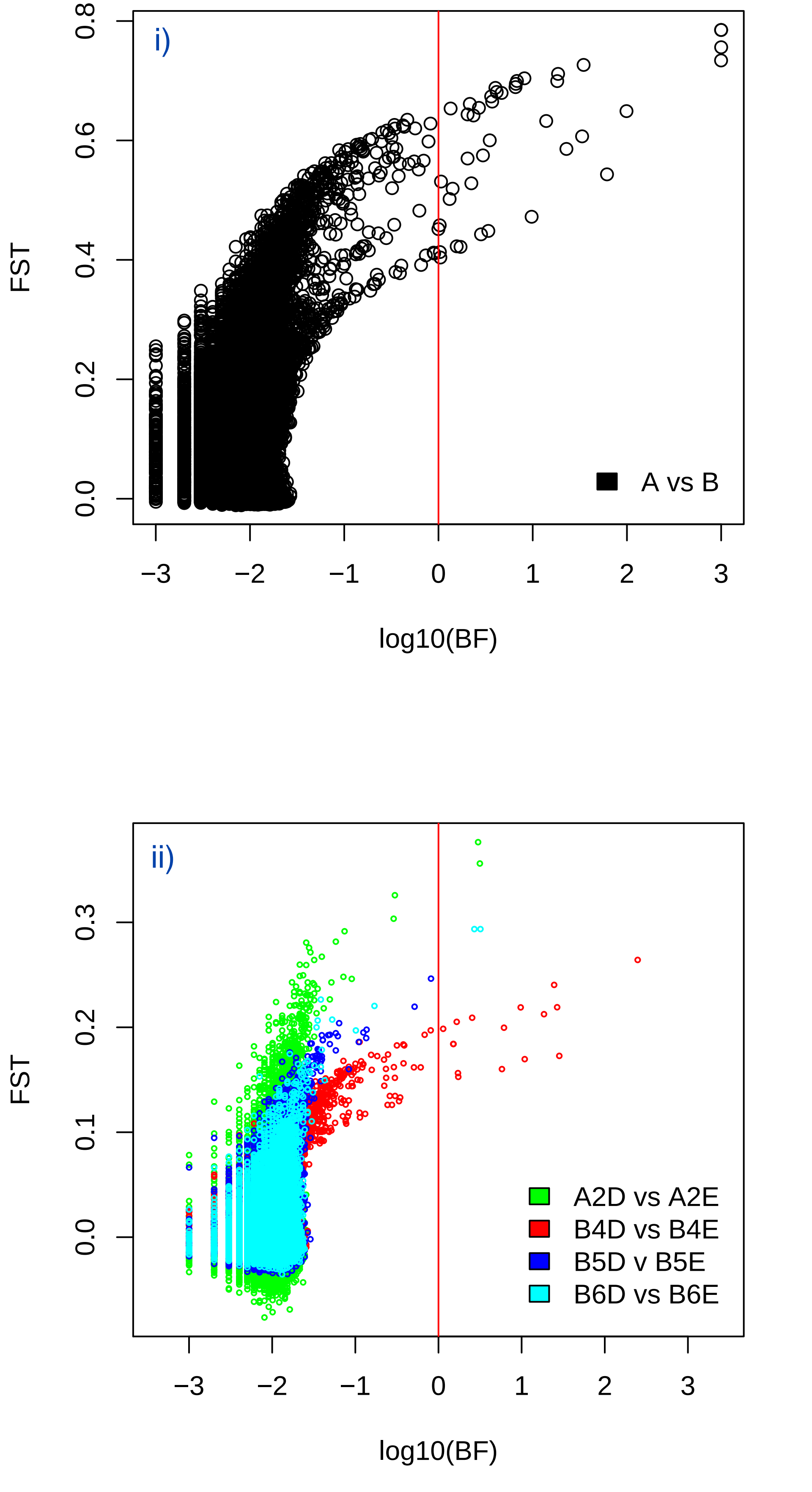

Supplement: Supplementary file 9 — Fig S9 [file EVA-13-1145-s009.pdf]
