# Supplementary material for: Demographic history and genomics of local adaptation in blue tit populations
Source: Evol Appl. 2020 Jul 14;13(6):1145–65. doi: 10.1111/eva.13035 (PMC7359843; doi:10.1111/eva.13035)

Supplementary Figure 10. Histograms of SNP loadings for RDA i) constrained to test divergence between deciduous and evergreen populations and ii) constrained to test divergence between mainland and Corsica

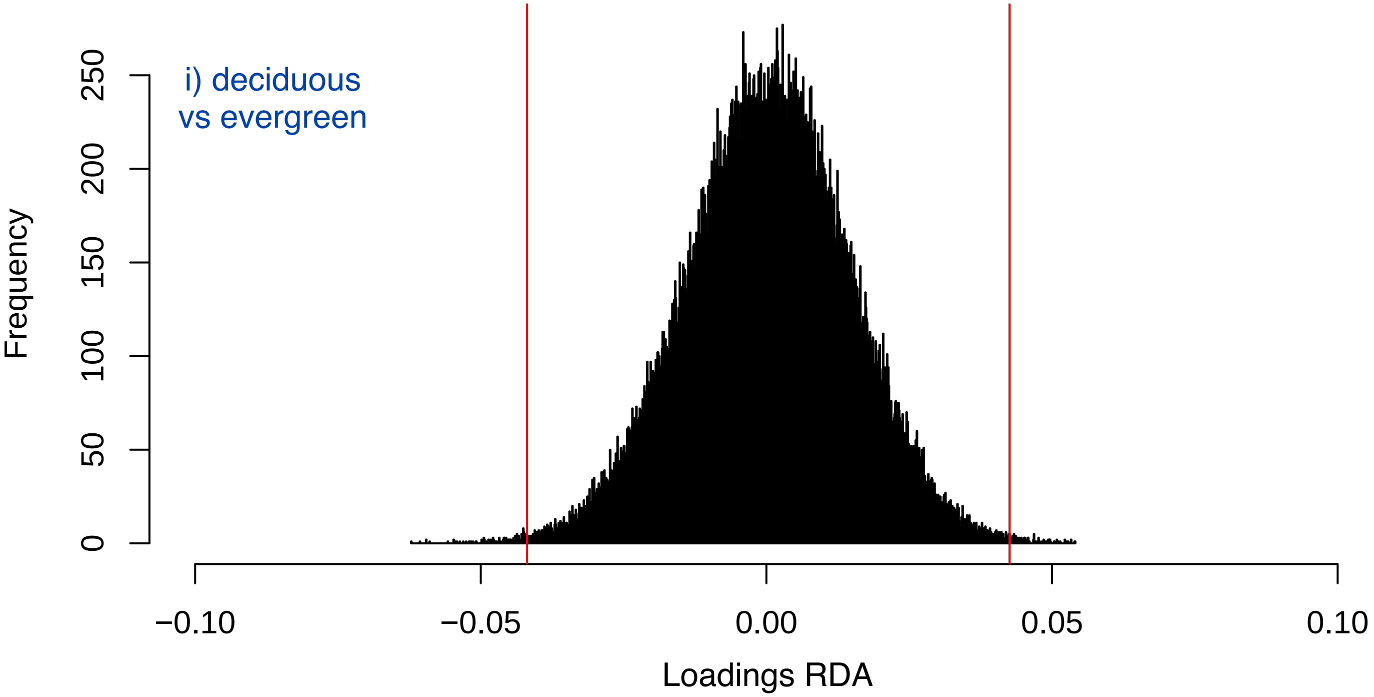

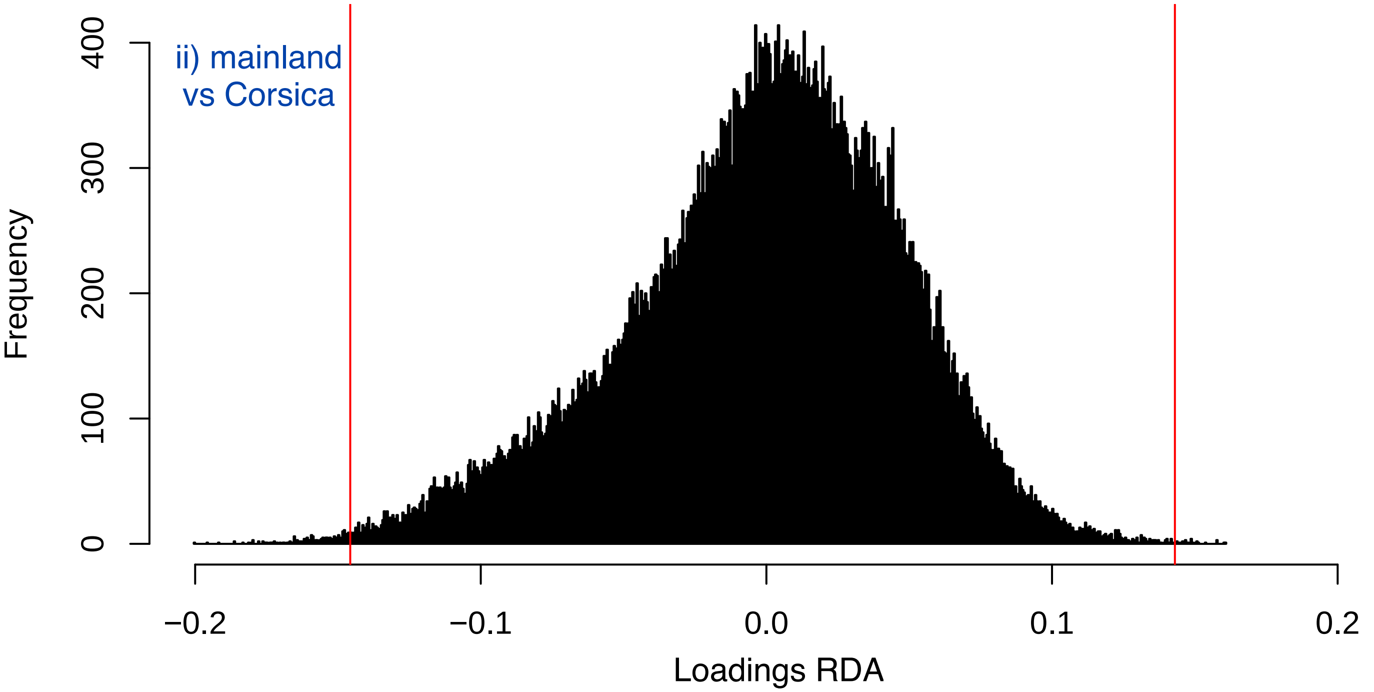

Supplement: Supplementary file 10 — Fig S10 [file EVA-13-1145-s010.pdf]
